# Unique combination of herbal ingredients for everyday distress in medical workers (short-term pilot study)

**DOI:** 10.1192/j.eurpsy.2022.1315

**Published:** 2022-09-01

**Authors:** M. Morozova, A. Alexeev, G. Rupchev, A. Beniashvili, S. Potanin, T. Lepilkina, D. Burminskiy

**Affiliations:** 1 FSBI Research Center of Mental Health, Laboratory Of Psychopharmacology, Moscow, Russian Federation; 2 Moscow State University, Faculty Of Psychology, Moscow, Russian Federation

**Keywords:** medical workers, herbal ingredients, Distress

## Abstract

**Introduction:**

Psychological distress is a phenomenon that often occurs not only in patients but in normal subjects under excessive psychological pressure. Health care workers are at particular risk of distress in a pandemic. It negatively affects the quality of life, social and physical functioning and can be a trigger of different diseases. The pharmaceutical drugs can be unnecessary active for healthy subjects. Nutraceuticals may be the adequate choice in this situation.

**Objectives:**

Assessing the effectiveness of the unique antistress combination of the three herbal ingredients (standardized extracts of passionfruit, melissa and catnip) in medical workers with the signs of psychological distress

**Methods:**

Twenty-four subjects-medical doctors from 30 to 55 years old (15 women; 9 men) were included into the one-week study. Antistress combination was administered 1 tablet tid. The first part of the State-Trait Anxiety Inventory (STAI “State anxiety”) andor a free self-report were done twice (before and at the end of the study)

**Results:**

From 24 subjects 19 subjects filled out the STAI, free self-reports were received from 10 subjects (5 people provided information about their condition in two forms). STAI scores showed statistically significant decrease in anxiety at the end of the study. A positive effect the emotional condition and quality of sleep was noted in free self-reports. Adverse effects of nutraceuticals were rare, mild, and transient. No negative impact on quality of working condition was registered.

**Conclusions:**

The pilot study showed the promising effect of antistress combination in medical workers in specific stressful situation.

**Disclosure:**

No significant relationships.

